# Diversity and environmental distribution of the cosmopolitan endosymbiont “*Candidatus* Megaira”

**DOI:** 10.1038/s41598-018-37629-w

**Published:** 2019-02-04

**Authors:** Olivia Lanzoni, Elena Sabaneyeva, Letizia Modeo, Michele Castelli, Natalia Lebedeva, Franco Verni, Martina Schrallhammer, Alexey Potekhin, Giulio Petroni

**Affiliations:** 1 Department of Biology, University of Pisa, Pisa, Italy; 2 Faculty of Biology, Saint Petersburg State University, Saint Petersburg, Russia; 3 Centro Romeo ed Enrica Invernizzi Ricerca Pediatrica, Dipartimento di Bioscienze, Università degli studi di Milano, Milan, Italy; 4 Core Facilities Centre “Culture Collections of Microorganisms”, Saint Petersburg State University, Saint Petersburg, Russia; 5 Microbiology, University of Freiburg, Freiburg, Germany

## Abstract

Members of the order *Rickettsiales* are often found in association with ciliated protists. An interesting case is the bacterial endosymbiont “*Candidatus* Megaira”, which is phylogenetically closely related to the pathogen *Rickettsia*. “*Candidatus* Megaira” was first described as an intracellular bacterium in several ciliate species. Since then it has been found in association with diverse evolutionary distantly-related hosts, among them other unicellular eukaryotes, and also algae, and metazoa, such as cnidarians. We provide the characterization of several new strains of the type species “*Candidatus* Megaira polyxenophila”, and the multidisciplinary description of a novel species, “*Candidatus* Megaira venefica”, presenting peculiar features, which highlight the diversity and variability of these widespread bacterial endosymbionts. Screening of the 16S rRNA gene short amplicon database and phylogenetic analysis of 16S rRNA gene hypervariable regions revealed the presence of further hidden lineages, and provided hints on the possibility that these bacteria may be horizontally transmitted among aquatic protists and metazoa. The phylogenetic reconstruction supports the existence of at least five different separate species-level clades of “*Candidatus* Megaira”, and we designed a set of specific probes allowing easy recognition of the four major clades of the genus.

## Introduction

The family *Rickettsiaceae* (*Rickettsiales*, *Alphaproteobacteria*) comprises obligate intracellular bacteria hosted by eukaryotic organisms. *Rickettsia* and *Orientia* are the most studied genera and they are etiological agents of many human diseases, such as spotted fever or typhus^1^. Within the *Rickettsiaceae* family, several other bacteria are not of direct health concern and have been also referred to as “neglected *Rickettsiaceae*”^2^. These bacteria are important for understanding key evolutionary and ecological phenomena, such as host-symbiont interactions^3,4^, the emergence of pathogenicity^5,6^, and even the evolution of mitochondria, which are considered by several authors related to *Rickettsiales*^7^.

Traditionally, members of the *Rickettsiaceae* family were known to colonize only arthropods and vertebrates^8^, but with the discovery and characterization of new species and genera phylogenetically affiliated to this family, this conviction vanished. Indeed, recent studies^2,9–14^ unraveled the existence of many “neglected *Rickettsiaceae*”, often hosted by protists. Considering the interwoven phylogenetic relationships among bacteria harbored by diverse hosts^15^, these data provided indirect evidence that some *Rickettsiaceae* can be frequently transmitted among hosts from evolutionary far related lineages^9^.

An interesting bacterial genus within “neglected *Rickettsiaceae*” is the endosymbiont “*Candidatus* Megaira”^2^. The first molecular report of this endosymbiont was in 2005 from the ciliate *Diophrys oligothrix*, where it was mentioned as a member of *Rickettsiaceae*^16^. Subsequently, this bacterium was found in many different hosts ranging from multicellular organisms, like cnidarians^17,18^ to green algae including chlorophytes and streptophytes^19–21^, and protists - amoebae^11^, and numerous ciliates^2,12,22^. In some cases, bacteria were associated to the gut content of animals, such as ascidians^23^, fish^24^, or worms^25^. As these studies dealt mainly with molecular approaches, the true nature of these associations was not clarified. Some full-length 16S rRNA gene sequences belonging to the genus “*Ca*. Megaira” have been retrieved from environmental samples, such as freshwater lakes^26^, aquaria^27^, wastewater treatment plants^28^, and soil^29^, but these studies do not allow conclusions regarding their association with possible hosts.

Notably, the type species “*Ca*. Megaira polyxenophila” was retrieved in diverse species of ciliates belonging to different classes^2^, including the fish parasite *Ichthyophthirius multifiliis*^12^, and in a wide range of additional, evolutionary distant, organisms^11,17–21^. In ciliates, this bacterial endosymbiont is able to colonize different host cell compartments (e.g. macronucleus or cytoplasm) according to the host species. Colonization of ciliate cells by bacteria can be tentatively explained accounting that these protists, as phagotrophic predators, can engulf bacteria, which may escape from digestion and colonize one of many cell compartments available^30,31^. Considering that ciliates and other protists are at the basis of many trophic chains^32^, it is tempting to hypothesize that they may transmit their symbionts to other hosts at higher trophic levels^2^.

Herein we report 14 new strains of “*Ca*. Megaira polyxenophila” and describe the novel species “*Ca*. Megaira venefica”. We also provide a critical revision of the genus “*Ca*. Megaira”, the already described species, and new clades it comprises. We designed and validated a new genus-specific probe for fluorescence *in situ* hybridization (FISH) experiments in order to allow an easy recognition of members of the genus. Moreover, we designed *in silico* four clade/species specific probes targeting the same hypervariable 16S rRNA region, and validated two of them for which strains of “*Ca*. Megaira polyxenophila” and “*Ca*. Megaira venefica” were available. Screening of 16S rRNA gene amplicons applying the Integrated Microbial Next Generation Sequencing (IMNGS)^33^ platform demonstrates the high diffusion of this bacterial genus in many ecosystems.

## Results

### Host identification

A preliminary screening for the presence of bacterial symbionts was performed by Differential Inference Contrast (DIC) microscopy on paramecia residing in the Core Facilities Centre “Culture Collections of Microorganisms”. When a bacterial symbiont was detected, usually all *Paramecium* hosts were infected and then molecularly characterized. A list of ciliate cultures harbouring bacterial endosymbionts affiliated to “*Ca*. Megaira” is provided (Table 1). As for the host species not present in the culture collection, infection of “*Ca*. Megaira” was recorded only in some of the isolated ciliates (e.g. *Colpidium striatum* ASP_B; *Paramecium caudatum* RFL1, RanNy1602-AP18 and Mue14b).

**Table 1:** List of ciliate cultures harbouring bacterial endosymbionts.

Symbiont	Host	Culture index	Intracellular localization	Origin	Sampling geographical coordinates
“*Candidatus* Megaira polyxenophila”	*Colpidium striatum*	ASP_B (polyclonal)	Cytoplasm	Italy, Perugia (fish-farm)	42.763018, 12.861376
	*Paramecium primaurelia*	ThK-1 (monoclonal)	Cytoplasm	Thailand, Phi-Phi Don islands, (stream)	7.737428, 98.773924
	*Paramecium primaurelia*	IP 4-1 (monoclonal)	Cytoplasm	Italy, Pisa (drain)	43.432147, 10.224717
	*Paramecium primaurelia*	IP 17-21 (monoclonal)	Cytoplasm	Italy, Pisa (drain)	43.441793, 10.225463
	*Paramecium pentaurelia*	Nr1-10 (monoclonal)	Cytoplasm	Russia, Novorossijsk (wastewater)	44.423800, 37.470800
	*Paramecium caudatum*	NV 2-5 (monoclonal)	Macronucleus	Russia, Velikiy Novgorod, (pond)	58.311554, 31.161758
	*Paramecium caudatum*	Sp 11-8 (monoclonal)	Macronucleus	Spain, Madrid, (drain)	40.244900, 3.410472
	*Paramecium caudatum*	Sp 9-41 (monoclonal)	Macronucleus (presence of *Holospora undulata* in micronucleus)	Spain, Madrid, (pond)	40.250417, 3.410606
	*Paramecium caudatum*	Sp 9-5 (monoclonal)	Macronucleus (presence of betaproteobacterium in cytoplasm)	Spain, Madrid, (pond)	40.250417, 3.410606
	*Paramecium caudatum*	Sp 9-22 (monoclonal)	Macronucleus (presence of betaproteobacterium in cytoplasm)	Spain, Madrid, (pond)	40.250417, 3.410606
	*Paramecium caudatum*	VL 10-1 (monoclonal)	Macronucleus	Russia, Vladimir, (wastewater pond)	56.101171, 40.434921
	*Paramecium caudatum*	RFL1 (polyclonal)	Macronucleus	Russia, Ropsha (fish-farm)	59.725603, 29.858563
	*Paramecium caudatum*	RanNy1602-AP18	Macronucleus	Germany, Rangsdorf (lake)	52.289597, 13.407075
	*Paramecium caudatum*	Mue14b	Macronucleus	Germany, Muenster (canal)	51.968526, 7.632249
“*Candidatus* Megaira venefica”	*Paramecium bursaria*	1M-2^(T)^ (monoclonal)	Cytoplasm	Russia, Peterhof (wastewater drain)	59.879020, 29.851318
	*Paramecium bursaria*	VL 3-1 (monoclonal)	Cytoplasm	Russia, Vladimir (pond)	56.159550, 40.360211
	*Paramecium bursaria*	VL 12-10 (monoclonal)	Cytoplasm	Russia, Vladimir (pond)	56.157310, 40.357658
	*Paramecium nephridiatum*	Sr 2-6 (monoclonal)	Cytoplasm	Russia, White sea (brackish pool)	66.287654, 33.666561
	*Paramecium putrinum*	ETu 7-4 (monoclonal)	Cytoplasm	Estonia, Tartu (canal)	58.369089, 26.751568

The traits used for species recognition within ciliate genera should be considered reliable only if they diverge sufficiently from one species to another one, and are considered as a whole and not individually^34^. With this perspective, in this study, ciliates were identified up to morphospecies level, combining morphological investigation on features, such as the cell shape, size, and micronuclear appearance and number (data not shown) with molecular characterization through 18S rRNA gene sequencing (for further details see Supplementary Table S1). In case of hosts belonging to the *Paramecium aurelia* complex, sequencing of cytochrome oxidase *c* subunit I (COI) gene and internal transcribed spacer (ITS1-5.8S-ITS2) was also performed to identify the species (Supplementary Table S1).

18S rRNA gene sequence of algal endosymbionts of *Paramecium bursaria* strains was 99.6% identical to *Micractinium condutrix* (Accession Number KF887344).

### Molecular characterization of bacterial endosymbionts

#### 16S rRNA gene sequencing and sequence comparison

The majority of the nearly full-length 16S rRNA gene sequences characterized in this study displayed identity higher than 99.7% with “*Ca*. Megaira polyxenophila” (Supplementary Table S2), and were consequently assigned to this species. In case of *P*. *caudatum* Sp9-41, a second symbiont morphologically similar to *Holospora undulata* was present in the micronucleus (Supplementary Fig. S1). Its 16S rRNA gene (accession number MG563930, 1404 bp) was obtained after cloning, and confirmed the assignment to *H*. *undulata*.

The sequences of the novel “*Ca*. Megaira” species inhabiting the cytoplasm of *P*. *bursaria*, *P*. *nephridiatum* and *P*. *putrinum* strains were 1413 bp long and shared the highest identity with members of the “*Ca*. Megaira” Clade C, namely uncultured *Rickettsiales* bacteria obtained from *Hydra oligactis*, several uncultured bacteria associated to *Ichthyophthirius multifiliis*^12^, and bacterial sequences retrieved from environmental samples of lakes and soil (range 98.6–99.8%, Supplementary Table S3). The newly characterized sequences presented few nucleotides of difference between symbionts from different host species (Supplementary Table S3).

16S rRNA gene sequence identities were calculated for all members of the genus “*Ca*. Megaira” (Supplementary Table S4), and confirmed the existence of at least five clades within the genus.

A critical manual inspection of available sequences from NCBI nucleotide database showed the presence of 9 previously unrecognized chimeras, and 20 misclassified sequences. Sequences were firstly screened with nucleotide BLAST; if identity was more pronounced only on one side of the sequence, this was split in two parts in the supposed breaking region. The two sequence parts were independently blasted, and if only one of them provided BLAST results similar to the whole sequence, the other one being significantly different from the best hit, the original sequence was considered a chimera and consequently reannotated. Four identified chimeras were particularly hard to detect, as they originated from two diverse “*Ca*. Megaira” clades (AM159487; HQ691997; KT851814; KT851825). This observation suggests that representatives of two “*Ca*. Megaira” clades were co-occurring in the same host/sample. Some of these chimeras have been included in previous phylogenies of the genus (e.g. AM159487; HQ691997; KT851814; KT851825); these sequences were excluded from the following analyses (Supplementary Table S5) and their putative chimeric origin has been communicated to GenBank to implement public annotation. In our strains, we detected the presence of a single “*Ca*. Megaira” species at once. On the contrary, a critical screening of “Ca. Megaira” sequences retrieved from different *I*. *multifiliis* strains evidenced sometimes the presence of representative from two “*Ca*. Megaira” clades, namely Clade A and Clade E in the same host. Indeed, in our chimera screening, in these hosts we also detected chimeric sequences between these two clades.

In accordance with previous studies^2^, members of Clade A “*Ca*. Megaira polyxenophila” showed identity values above 98.6%. Members of Clade D had 99.2% of identity among themselves, while they shared identity values between 97.3% and 98.5% with Clade A (when only full-length sequences were considered), and these values are below the threshold established (98.65-98.7%) to discriminate different bacterial species according to 16S rRNA gene sequence^35^. The situation of Clade B was more heterogeneous; indeed, all members shared a minimum identity of 96.5%, which may reveal further inner species level subdivision. On the contrary, Clade C and Clade E were more homogeneous and had identity range higher than 98.6% and 99.5%, respectively. Identities of members of Clade C with other “*Ca*. Megaira” clades did not exceed 98.3%. Therefore, the newly characterized symbionts together with previously available sequences allowed to define Clade C as a new species, which we named “*Ca*. Megaira venefica” (a formal description is provided as Supplementary Text S1).

### Phylogenetic analyses

**Figure 1:**
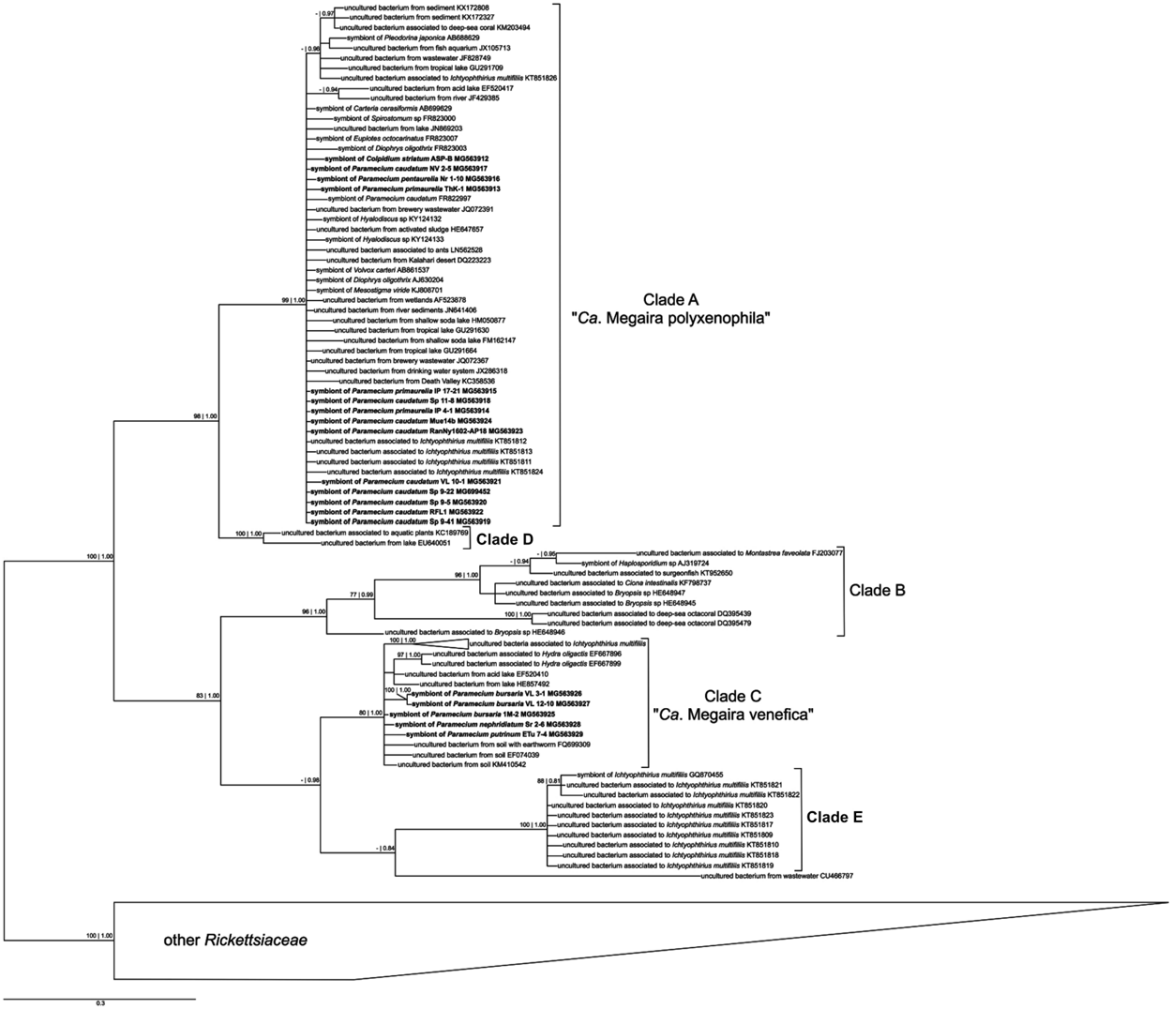
Bayesian inference phylogenetic tree of the genus “*Candidatus* Megaira” based on 16S rRNA gene sequences inferred with the GTR + I + G model. Numbers associated to each node represent bootstrap values inferred after 1000 maximum likelihood pseudo-replicates and Bayesian posterior probabilities (values below 70|0.70 are not shown). Sequences in bold were characterized in this study, and “*Ca*.” stands for “*Candidatus*”. The scale bar represents an estimated genetic distance of 0.3.

Our phylogenetic reconstruction of “*Ca*. Megaira” shows an updated scenario compared to previous studies^2,11,12^, where only up to four clades were reported. Indeed, we confirmed Clade A and B, which were retrieved by all previous authors^2,11,12^, we followed the suggestion by Zaila *et al*.^12^ to split Clade C of Schrallhammer *et al*.^2^ into two and named them respectively Clade C and E. The presence of an additional clade suggested by Hess^11^, named Clade D, was confirmed. Additionally, we identified one sequence from wastewater (CU466797), which is clearly not affiliated to any of the described clades, but instead forms a sister group of Clade E, thus suggesting the possible existence of an additional clade (Fig. 1). This hypothesis is also sustained by phylogenetic analysis on hypervariable regions presented below, where region V4–V6 of this sequence is embedded in a large clade. At variance with previous studies, we could recognize several published sequences as possible chimeras, and removed them from the phylogenetic analyses.

The clade comprising the type species “*Ca*. Megaira polyxenophila”, referred to as Clade A by previous authors^2,11,12^, is highly supported (99% bootstrap value for Maximum Likelihood (ML) and 1.00 of posterior probability), and includes all our characterized symbionts assigned to this species. Clade D, sister group of Clade A, is fully supported (100% bootstrap value for ML and 1.00 of posterior probability for BI), and consists of two sequences isolated from aquatic plants and a freshwater lake, respectively. Clade B is well supported (96% bootstrap value for ML and 1.00 of posterior probability for BI), and contains almost only bacteria associated to marine hosts, such as *Bryopsis*^19^, corals^18^, and a *Haplosporidium* species infecting a marine mollusc^36^. Clade C is moderately supported (80% bootstrap value for ML and 1.00 of posterior probability for BI), and includes the new species “*Ca*. Megaira venefica”, represented by symbionts found in *Paramecium* (current study), bacteria associated to *Hydra oligactis*, *I*. *multifiliis*, and by uncultured environmental bacteria. Finally, Clade E comprises exclusively bacteria detected in the fish parasite *I*. *multifiliis* and is highly supported (100% bootstrap value for ML and 1.00 of posterior probability for BI).

### Fluorescence *in situ* hybridization

**Figure 2:**
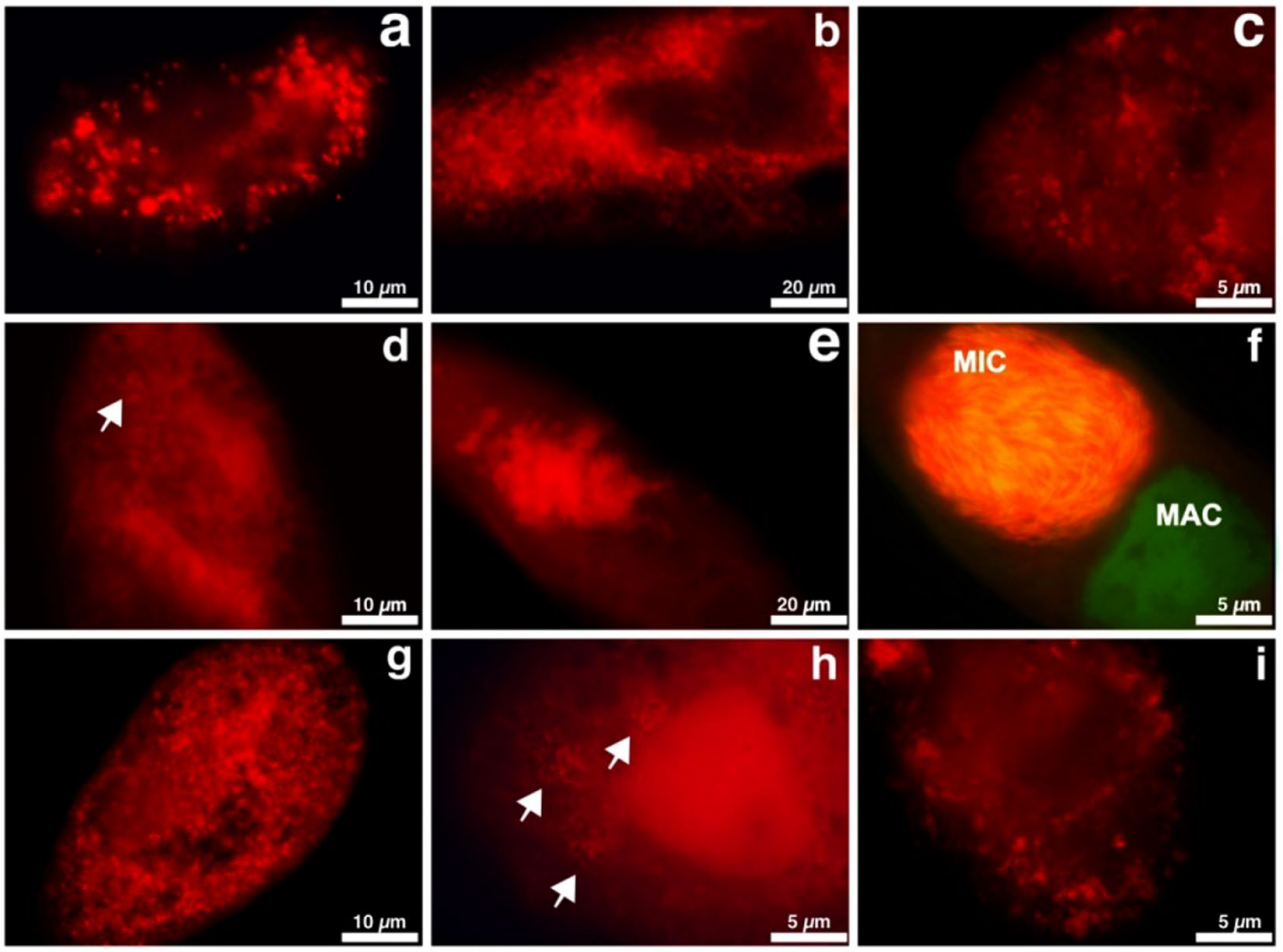
Fluorescence *in situ* hybridization of “*Candidatus* Megaira polyxenophila” and “*Candidatus* Megaira venefica”. “*Ca*. Megaira polyxenophila” targeted by probe MegPoly_66 (Cy3-labelled, red signal) from the cytoplasm of *Colpidium striatum* ASP-B (**a**), *P*. *primaurelia* ThK-1 (**b**), *P*. *primaurelia* IP 17-21 (**c**), *P*. *pentaurelia* Nr1-10 (**d**), and the macronucleus of *P*. *caudatum* Sp 11-8 (**e**). Double nuclear infection in *P*. *caudatum* Sp 9-41: *Holospora undulata* (Cy3-labelled specific probe for *Holospora*, red signal) in the micronucleus (MIC), and “*Ca*. Megaira polyxenophila” (MegPoly_66 probe labelled with fluorescein, green signal) in the macronucleus (MAC) (**f**). “*Ca*. Megaira venefica” in the cytoplasm of its respective hosts *P*. *bursaria* (**g**), *P*. *nephridiatum* (**h**), and *P*. *putrinum* (**i**), targeted by probe MegVene_95 (Cy3-labelled, red signal). White arrows indicate the presence of symbionts within the host cells.

One new genus-specific probe was designed for FISH experiments, which targeted the symbionts of interest with high specificity with one exception (Table 2): i.e. the probe designed for the genus (Megenus_487) matched *in silico* all available members of “*Ca*. Megaira” present in our phylogenetic reconstruction (Fig. 1) but also had a single unspecific hit with an uncultured gammaproteobacterium sequence (Table 2). Other organisms positive to probe Megenus_487 in Ribosomal Database Project (RDP)^37^ turned out to be misclassified “*Ca*. Megaira” sequences (often recorded as *Orientia*, or uncultured *Alphaproteobacteria*), or chimeric sequences (Supplementary Table S5). Additionally, four specific probes were additionally designed to recognize the most abundant clades A + D, B, C and E; all these probes showed a high *in silico* specificity for the targeted sequences with no unspecific hits. The hypervariable region chosen as target site was basically the same, and it was comprised between position 66 and position 95 according to *E*. *coli* 16S rRNA gene reference. Only two clade-specific probes (A + D, C) could be experimentally tested with FISH experiments using available strains (i.e. “*Ca*. Megaira polyxenophila”, and “*Ca*. Megaira venefica”) at different formamide concentrations (0%, 15%, and 30%). For the other untested probes, we assumed that they should work properly as they were designed on the same 16S rRNA gene region, which should be accessible as in the tested closely related “*Ca*. Megaira” species. Nevertheless, future controls are recommended once strains of these clades will be available. The signal for the two tested probes resulted visible at all formamide levels, and signal intensity was optimal at 0% formamide (Fig. 2).

**Table 2:** *In silico* matching of “*Candidatus* Megaira” probes against bacterial 16S rRNA gene sequences available from RDP (release 11, update 4) and SILVA (release 123) databases.

Probe name	Target	Sequence	RDP	SILVA
Megenus_487	All genus members	5′-GCCGGGGCTTTTTCTGTTGGT-3′ (T_m_ = 61.8 °C)	1	0
MegPoly_66	“*Candidatus* Megaira polyxenophila” Clade A and “*Candidatus* Megaira” Clade D	5′-GCAAGCCCCAATTTTGTTCGT-3′ (T_m_ = 57.9 °C)	0	0
MegairaB_76	“*Candidatus* Megaira” Clade B	5′-YCTGAAGCAAGCTCCAGC-3′ (T_m_ = 57.1 °C)	0	0
MegVene_95	“*Candidatus* Megaira venefica” Clade C	5′-CCGTTTGCCACTAACGAC-3′ (T_m_ = 56.0 °C)	0	0
MegairaE_69	“*Candidatus* Megaira” Clade E	5′-GGTGCTTCGTCCAAAGGCATC-3′ (T_m_ = 61.8 °C)	0	0

The reported numbers indicate the number of non-specific target sequences detected by the probe against the total number of sequences matching the probe with 0 mismatches.

“*Ca*. Megaira polyxenophila” strains were located either in the cytoplasm in case of *Colpidium striatum* and members of the *P*. *aurelia* complex, or in the macronucleus in case of *P*. *caudatum* (Fig. 2a–e). Infection level and prevalence (number of infected individuals) was usually very high in each culture screened. In *P*. *pentaurelia* Nr1–10, we observed fluctuations in the number of symbionts per cell, ranging from only few bacteria per cell to densely filled ciliates. In three strains of *P*. *caudatum*, other intracellular bacteria were detected: strain Sp 9–41 harbored *H*. *undulata* in its micronucleus (Fig. 2f), while strains Sp 9-5 and Sp 9-22 hosted in their cytoplasm a betaproteobacterium, which is presently under investigation (manuscript in preparation, data not shown). *H*. *undulata* and the betaproteobacterium were not labelled by the employed “*Ca*. Megaira” probes at any formamide concentration. All *P*. *bursaria* strains and *P*. *putrinum* harboring “*Ca*. Megaira venefica” hosted symbionts homogeneously distributed in the cytoplasm (Fig. 2g,i), while cytoplasmic symbionts of *P*. *nephridiatum* clustered together in small groups (Fig. 2h). A further FISH control was performed using “*Ca*. Megaira polyxenophila” and “*Ca*. Megaira venefica” in the same experiment, and probes did not cross-react, thus showing good specificity (data not shown).

### Morphological and ultrastructural description of endosymbionts “*Candidatus* Megaira polyxenophila”

**Figure 3:**
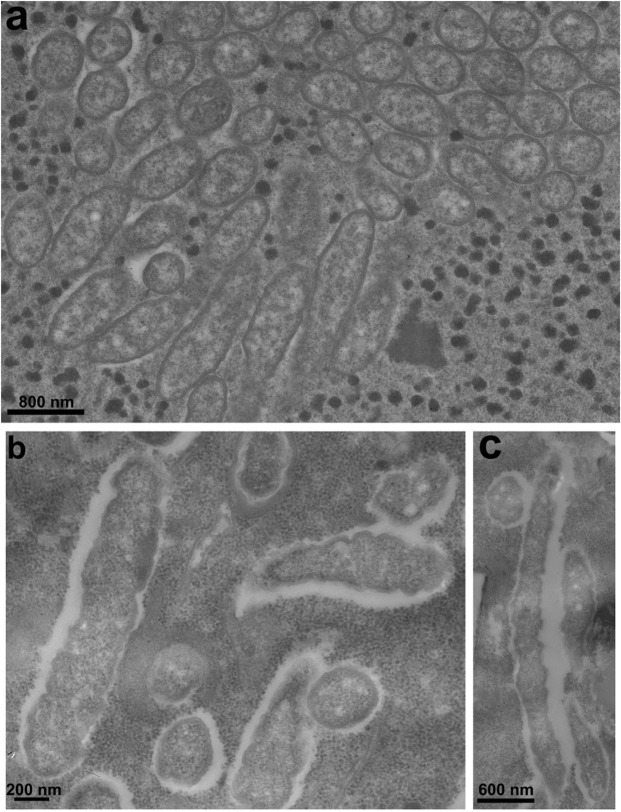
Transmission electron microscopy of “*Candidatus* Megaira polyxenophila”. View of macronuclear infection of “*Ca*. Megaira polyxenophila” in *P*. *caudatum* Sp 9-5 (**a**), longitudinal and transverse sections of “packed” “*Ca*. Megaira polyxenophila” in the cytoplasm of strain *P*. *primaurelia* ThK-1 (**b**), longitudinal and transverse sections of “giant” endosymbionts found in the cytoplasm of strain *P*. *primaurelia* ThK-1 (**c**).

All “*Ca*. Megaira polyxenophila” strains studied presented the classical “*Ca*. Megaira polyxenophila” ultrastructure^2,21^, consisting of double membrane (typical of Gram-negative bacteria), homogeneous cytoplasm, a clear halo surrounding the cells, and size dimensions in the range (~1.5 µm × 0.6 µm). Cytoplasm of *P*. *primaurelia* strain ThK-1 and macronucleus of *P*. *caudatum* strain Sp 9-5 were densely packed with bacteria (Fig. 3a,b), but also some bacteria-free space was always left. Some bacterial cells from the cytoplasm of strain ThK-1 appeared “giant” when compared to previous descriptions of “*Ca*. Megaira polyxenophila”, reaching length of ~4.8 µm (Fig. 3c).

### Novel “*Candidatus* Megaira” species: “*Candidatus* Megaira venefica”

**Figure 4:**
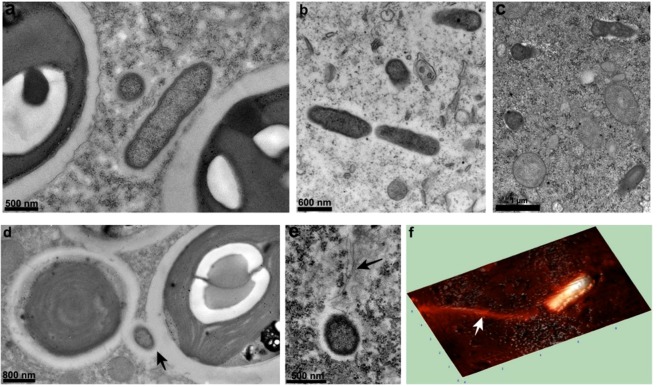
Ultrastructure of “*Candidatus* Megaira venefica”. TEM images of two “*Ca*. Megaira venefica” cells in the cytoplasm of *P*. *bursaria* strain 1M-2^(T)^, shown in transverse and longitudinal sections (**a**). Endosymbionts in the cytoplasm of *P*. *putrinum* (**b**), and in *P*. *nephridiatum* (**c**). “*Ca*. Megaira venefica” associated to the perialgal vacuole (black arrow) in *P*. *bursaria* strain 1M-2^(T)^ (**d**), cell of “*Ca*. Megaira venefica” reporting flagella (black arrow) detected in the cytoplasm of *P*. *bursaria* strain VL 12-10 (**e**). Atomic force microscopy image of “*Ca*. Megaira venefica” in strain VL 12-10, showing the putative flagellum (white arrow; **f**).

Symbionts identified as the novel “*Ca*. Megaira” species appeared rod-shaped and presented a typical Gram-negative ultrastructure consisting of two membranes with a homogeneous cytoplasm. The length of bacteria ranged from ~1.9 µm in *P*. *bursaria* to 1.4 µm in *P*. *putrinum*, while width was constantly 0.4 µm. Bacteria were mostly present in the ciliate cytoplasm, but their abundance varied according to the *Paramecium* species: symbionts in *P*. *bursaria* and in *P*. *putrinum* were scattered, whereas in *P*. *nephridiatum* they were grouped together in rare clusters (Fig. 4a–c).

In *P*. *bursaria*, half of bacteria were also found closely associated to the membrane of perialgal vacuole containing symbiotic algae symbionts of *P*. *bursaria* (Fig. 4d). The possible localization of bacteria inside the periagal membrane was detected only once, so we cannot rule out the possibility of fixation artefacts as well (Supplementary Fig. S2). However, bacteria were never noticed to invade the symbiotic algal cells. Generally, no pili and flagella were observed, except for the strain VL 12-10, where structures resembling flagella were occasionally detected (Fig. 4e,f).

### Diversity and environmental distribution of “*Candidatus* Megaira”

**Figure 5:**
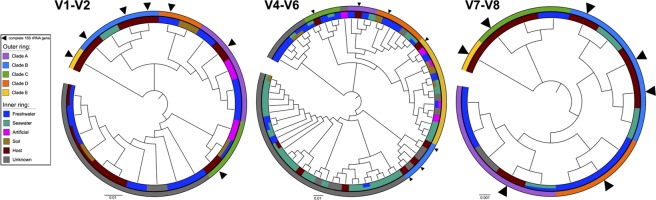
Diversity of “*Candidatus* Megaira” based on 16S rRNA gene amplicon search in IMNGS. Phylogenetic trees of different 16S rRNA gene hypervariable regions. IMNGS hits longer than 300 bp were clustered in OTUs with 99% identity and separated for each hypervariable region taken into analysis. A total number of 194 OTUs were obtained: 42 for V1–V2, 135 for V4–V6, 17 for V7–V8. Complete 16S rRNA gene sequences were employed in the analysis to enlighten the diversity of each “*Ca*. Megaira” clade. OTUs were also assigned to environmental compartments according to the sequence origin (outer ring).

**Figure 6:**
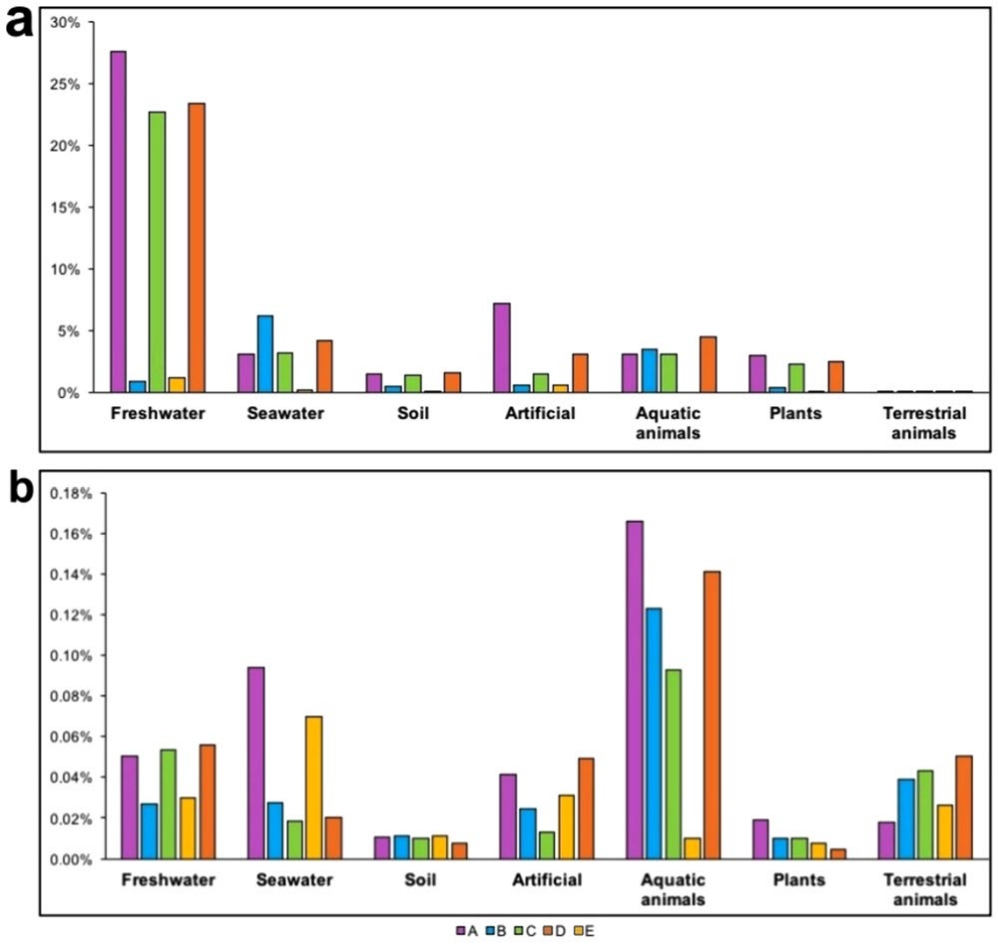
Environmental distribution of “*Candidatus* Megaira” based on 16S rRNA gene amplicon search in IMNGS. Frequency of occurrence estimated as the number of times with which “*Ca*. Megaira” occurred in all samples. On the x axis the categories of environment and host samples are represented, whereas in the y axis the percentage of frequency of occurrence is shown (**a**). Relative abundance of “*Ca*. Megaira” expressed as the ratio between “*Ca*. Megaira” positive samples and the total number of samples for a certain environment or host. On the x axis the categories of environment and host samples are represented, whereas in the y axis the percentage of relative abundance is shown (**b**).

“*Ca*. Megaira” diversity and environmental distribution in previous metabarcoding studies was examined using IMNGS platform^33^. The output of IMNGS similarity search with a threshold of 95% identity with “*Ca*. Megaira” sequences produced a total number of 11373 centroid sequences (identity higher than 99%, as by IMNGS default settings) originating from 4673 samples. After removal of short and low quality sequences, all remaining 11306 sequences were pooled and re-clustered in 194 OTUs using a 99% identity threshold. OTUs were divided in three different groups according to which hypervariable region of 16S rRNA gene they belonged to and phylogenetic trees were constructed for each hypervariable region. In all trees, the five major clades of “*Ca*. Megaira” are distinguishable and their relationships are in agreement with full-length 16S rRNA gene phylogeny: Clade A and D are sister groups, as well as Clade C and Clade E, whereas Clade B is always separate (Fig. 5). However, many not yet described lineages are present in addition to the previously identified clades, for example in the tree based on hypervariable region V4–V6, at least six additional clades can be observed (Fig. 5). It was impossible to assign each clade to a specific environment according to OTU environmental distribution (Fig. 5), thus a more detailed analysis was performed to investigate the ecology of different “*Ca*. Megaira” clades. Frequency of occurrence (calculated as the number of microbiome samples positive for “*Ca*. Megaira” divided the total number of samples of each category) and relative abundance (determined as the number of “*Ca*. Megaira” reads divided total number of reads for each sample) of the five “*Ca*. Megaira” clades were estimated for environments and host categories (Fig. 6, for further details see Methods). Clades A, C, D had maximal frequencies of occurrence in freshwater samples reaching more than 20% (Fig. 6a), whereas their frequencies of occurrence were below 5% for the rest of environmental categories and less than 1% in terrestrial animals (Fig. 6a). On the other side, Clade B showed an overall lower occurrence, in particular, it was much less frequently retrieved in freshwater (less than 1% samples), while it had the highest frequency of occurrence in seawater (about 7%) (Fig. 6a). Clade E seemed to be very rare in the environment, thus lacking any significant tendency (Fig. 6a). All “*Ca*. Megaira” clades, all except Clade E, had a higher relative abundance in aquatic animals (more than 0.09%). In most cases, relative abundances were comprised between 0.01% and 0.06% including also environments where bacteria had a high frequency of occurrence (e.g. Clades A, C, D, in freshwater) or in in which they had a minimal frequency of occurrence (e.g. all clades in terrestrial organisms). Clade E showed very low relative abundances in all categories considered (Fig. 6b) except seawater, where it reached 0.06%; similarly, Clade A that has a low frequency of occurrence in seawater showed in the same environment a relatively high abundance of 0.09%.

## Discussion

The study presents the description of a novel “*Ca*. Megaira” species and characterization of new “*Ca*. Megaira polyxenophila” strains, together with screening of “*Ca*. Megaira”-related sequences in environmental 16S rRNA gene amplicons databases, providing further understanding of “*Ca*. Megaira” wide host range adaptation.

The new species “*Ca*. Megaira venefica” has been found in several strains of *Paramecium* isolated from different Russian and Estonian locations. Bacterial cells were usually located in the cytoplasm of their hosts, and, in case of *P*. *bursaria* and *P*. *putrinum*, symbionts were extremely numerous (Fig. 2). An interesting feature of bacteria found in *P*. *bursaria* strains was the predilection for being closely associated to the endosymbiotic algae inhabiting the ciliate cytoplasm (Fig. 4d; Supplementary Fig. S2). A similar “behavior” has been already reported for “*Ca*. Sonnebornia yantaiensis”, another cytoplasmic bacterial symbiont of *P*. *bursaria*^38^. Probably, bacterial endosymbionts benefit from this intracellular localization, as they find protection from host auto-digestion^31^, and at the same time, they may exploit nutrients and metabolites synthesized and eventually secreted by the endosymbiotic algae^39^.

The novel “*Ca*. Megaira” species displays morphological diversity, as some bacteria showed the presence of flagella (Fig. 4c,d). Some members of the order *Rickettsiales* possess either flagella or a set of flagellar genes^5,10,40–42^, and the presence of flagella is known to be an ancient trait of *Alphaproteobacteria*^43^, lost in many *Rickettsiales* probably due to adaptation to intracellular lifestyle. The maintenance of flagella/flagellar genes has been suggested to be involved in the infection process, symbiosis preservation, and transmission of the symbiont to other hosts, either as a motility organelle or as a type III secretion system^40,41,44^. Since flagella in “*Ca*. Megaira venefica” are not always present, it is tempting to hypothesize a connection with some unknown life-cycle stage. For example, flagella might be useful to colonize new hosts, possibly in conjunction with other players involved in molecular interactions, such as a type IV secretion system and a rOmbB-like surface protein, which have been detected in “*Ca*. Megaira polyxenophila” from green algae, and are putatively involved in pathogenesis in other *Rickettsiaceae*^21^.

Our ultrastructural investigations revealed that members of the “*Ca*. Megaira” genus possess variability even in cell dimensions. While the usual recorded cell size of “*Ca*. Megaira polyxenophila” was 1.0–1.6 µm in length and 0.3–0.4 µm in width^2,16,21,45^, in this work, the giant symbionts detected in the cytoplasm of *P*. *primaurelia* strain ThK-1 were almost three times longer (Fig. 3c). Cell size of “*Ca*. Megaira venefica” varied among strains from different *Paramecium* host species, as *P*. *nephridiatum* and *P*. *putrinum* symbionts were smaller than those of *P*. *bursaria*. Variation in cell size is known for bacteria from the order *Rickettsiaceae*, for example different size has been described within the same *Rickettsia* species^46^.

In ciliates screening studies, the most frequently found species of the genus is “*Ca*. Megaira polyxenophila”, which is able to colonize diverse hosts, and shows only limited intraspecies variation in the 16S rRNA gene. We characterized several strains of this bacterial symbiont both from novel hosts like *Colpidium striatum* and species of the *P*. *aurelia* complex, and from already known hosts such as *P*. *caudatum*. Our investigation supported previous studies^2^ showing that this symbiont displays cellular compartment specificity according to the host. In particular, “*Ca*. Megaira polyxenophila” inhabits the cytoplasm of the *P*. *aurelia* species, while in *P*. *caudatum* this symbiont was always found in the macronucleus (Table 1). Thus, even in the closely related hosts this bacterium demonstrates variability of interaction, probably due to peculiarities of the host biology: in *P*. *aurelia* the macronucleus is not a stable compartment being frequently resorbed in autogamy, while in *P*. *caudatum* autogamy is absent^47^.

Our sequence comparison analysis and phylogenetic reconstruction allows drawing a more defined scenario within the genus “*Ca*. Megaira” with respect to the previous studies^2,11,12^. Clade D is phylogenetically positioned as a different species from Clade A with high support (Fig. 1), despite data on this clade are still scarce, as only two complete 16S rRNA gene sequences are available so far, thus only hinting at the real variability within this clade. However, phylogenetic analysis of hypervariable regions discloses a much wider diversity of Clade D (Fig. 5). Clade B is highly supported in 16S rRNA gene phylogeny (Fig. 1). Nevertheless, its members displayed relatively high genetic diversity (lowest identity 96.5%, Supplementary Table S4), thus allowing to divide this clade into at least three separate sub-groups. 16S rRNA gene hypervariable region phylogenies confirm even greater diversity within the Clade B lineage, as many different OTUs are present within the single compact clade (Fig. 5). All these data taken together suggest that diversity of Clade B resides mainly in hypervariable regions. In any case, we have to take into account that part of the internal variability present in each “*Ca*. Megaira” clade could be partly attributed to PCR errors derived from cloning procedures that could artificially determine lower identity values^48^. A critical analysis of 16S rRNA gene hypervariable region phylogenies points out the presence of at least ten “*Ca*. Megaira” species-level clades (Fig. 5). Moreover, the single “stand-alone” sequence (CU466797) evidenced formed a separate clade in hypervariable region V4–V6 tree (Fig. 5). This hypervariable region highlighted the existence of at least other five clades, possibly representing new species, for which full-length 16S rRNA sequences are still missing. Thus, we can consequently predict as a minimum ten species within “*Ca*. Megaira” genus, half of which currently lack considerable molecular data.

We failed to assign directly each clade of the 16S rRNA gene hypervariable region phylogenies to a certain environment or host by checking OTUs ecological origins. A more detailed environmental investigation of a 16S rRNA gene short amplicon database was applied considering frequencies of occurrence and relative abundances for each “*Ca*. Megaira” clade (Fig. 6). Frequency of occurrence analysis clearly showed a preference of Clades A, C, and D for freshwater environments, whereas Clade B displayed a preference for marine ones. On the contrary, Clade E was rarely detected in all environments, and it is possible to speculate that this could be related to a higher host specificity of Clade E bacteria. Indeed, up to now, full-length sequences have been found exclusively associated to the fish parasitic ciliate *Ichthyophthirius multifiliis*^12^. Interestingly, when present, Clade E bacteria showed a relative abundance similar to those of other clades or, in seawater, even higher (up to 0.07%). In general, relative abundance, which is higher in aquatic animals, suggests that, in these samples, if present, “*Ca*. Megaira” is also relatively abundant. Intriguingly, this is true also for terrestrial animals in which “*Ca*. Megaira” is seldom found (frequency of occurrence <1%), but, when present, has relative abundance higher than in soil (i.e. the environment generally explored by terrestrial animals). The high relative abundance of clades A and E in seawater if compared to low frequency of occurrence could be explained as a specific abundance in unidentified microeukaryote present in those specific samples. Unfortunately, no reliable estimate is available on the potential presence of microeukaryotes existing in water samples used for these metabarcoding studies. In general, the comparison of data on frequency occurrence with those on relative abundance are coherent with the idea that “*Ca*. Megaira” is associated to eukaryotic hosts.

Nearly all studies reporting “*Ca*. Megaira” presence dealt with aquatic environments, and these bacteria were reported as endosymbionts of diverse hosts, both unicellular, namely ciliates^2,16,22^ and amoebae^11^, and multicellular ones, such as cnidarians^17,18^, and green algae^19^. Protozoa and unicellular green algae are known to be frequent hosts of “*Ca*. Megaira” symbionts^2^. However, these hosts are so far underrepresented in 16S rRNA gene metabarcoding studies and, interestingly, our results revealed the absence of “*Ca*. Megaira” in the few samples associated to unicellular organisms. At the same time, we strongly suppose that many positive environmental hits from aquatic samples or gut samples of aquatic organisms might be explained by presence of unnoticed protists hosting “*Ca*. Megaira” endosymbionts, especially considering that “*Ca*. Megaira” – like all *Rickettsiales* - has never been shown to have a free-living stage. On the contrary, terrestrial organisms’ samples are very numerous, but “*Ca*. Megaira” sequences were extremely rare in these datasets. Relative abundance of “*Ca*. Megaira” sequence is generally low, as it is an endosymbiotic bacterium and sampling procedures were not aimed to isolate only host organisms. The relative abundance of endosymbionts in ciliates can reach high values only in studies specifically designed to analyse the microbiome of single cells^49^. Although aquatic ecosystems appear to be preferential environments for these bacteria (Fig. 6a), our results show that also terrestrial plants (e.g. crops, *Arabidopsis thaliana*, tropical trees), or their associated microbiomes, are other ecological niches where “*Ca*. Megaira” can occur (Fig. 6b). This is also true for terrestrial animals where “*Ca*. Megaira” is rarely found (Fig. 6a), albeit significantly abundant in samples (Fig. 6b). Differently from ciliates’ endosymbionts, intracellular bacteria of metazoans are usually located only in some specific organ or tissue^50^. Up to now, the diversity of such ecosystems has probably not been sufficiently investigated, in particular concerning host species, but recent^23,51,52^ and ongoing studies of “terrestrial” hosts may contribute in widening our knowledge about transmission of this poorly known symbiont between different hosts and environments. Most of the known *Rickettsiales* genera are usually associated to aquatic organisms including protists^15^, which strongly suggests that the last *Rickettsiales* common ancestor was hosted by an aquatic eukaryote. The finding of some *Rickettsiales* species from different families hosted by arthropods^40,53^ indicates that this “terrestrial” shift of *Rickettsiales* has evolved independently several times in some lineages^4,15^. Consistently with this consideration, also “*Ca*. Megaira” could have been able to shift to “terrestrial” environment. Notably, protists are often associated to “*Ca*. Megaira”, and might serve as environmental vectors of these bacteria. Indeed, each of four major clades of “*Ca*. Megaira” has at least one representative found in symbiotic association with unicellular eukaryotes.

## Methods

### Hosts isolation, cultivation and identification

Several strains of ciliates were used in this study (Table 1). Ciliate cultures were maintained in lettuce medium inoculated with *Klebsiella aerogenes* at +18 °C (Sanyo climatic chamber). For *P*. *bursaria* illumination regime of 10 hours darkness and 14 hours light by 2000 lx lamps was used. Cultures are available and maintained at the RR CCM Core Facilities Centre “Culture Collections of Microorganisms” of St. Petersburg State University, Russia. Identification of ciliate species was performed morphologically^54^, and then confirmed by molecular analysis.

### DNA extraction and molecular characterization

Approximately 100 starved ciliate cells were repeatedly washed in sterile distilled water to minimize bacterial contamination, and then fixed in 70% ethanol. Total genomic DNA was extracted employing the NucleoSpin™ Plant II kit (Macherey-Nagel GmbH & Co., Dueren NRW, Germany) using the protocol for fungal DNA extraction.

Polymerase chain reactions (PCRs) were performed in a C1000™ Thermal Cycler (BioRad, Hercules, USA) with the high-fidelity TaKaRa Ex Taq (TaKaRa Bio, Inc., Otsu, Japan). All PCRs consisted of 35 cycles with a preliminary denaturation step at 94 °C for 3 min, then for each cycle denaturation at 94 °C for 30 seconds, annealing at 55 °C for 45 seconds and elongation at 72 °C for 90 seconds, and a final elongation step at 72 °C for 5 minutes^55^. PCR products were estimated through electrophoresis on 1% agarose, purified with EuroGold Cycle Pure Kit (EuroClone®, Milano, Italy), and then sequenced with appropriate internal primers at GATC Biotech (Cologne, Germany) (for further details see Supplementary Text S2).

### Phylogenetic analyses

The obtained 16S rRNA gene sequences were aligned automatically with the automatic aligner of the ARB software package^56^ using the SILVA database release 128, and then manually edited in order to optimize base-pairing in the predicted rRNA stem regions. A total number of 130 sequences were selected from the family *Rickettsiaceae* (118 belonging to “*Ca*. Megaira”, used as ingroup, and 12 others as outgroup). Firstly, 119 sequences longer than 1397 bp were used to build the character matrix, then 11 short sequences were added, and phylogeny was inferred.

The optimal substitution model was determined with jModelTest^57^ according to the Akaike Information Criterion. Phylogenetic analyses were performed using both ML and BI methods. ML tree was inferred with 1000 bootstrap pseudoreplicates using PhyML software 3.0^58^, while Bayesian Inference was carried out with MrBayes 3.2^59^ employing three runs, with one cold and three heated Monte Carlo Markov chains each, for 1000000 generations with a burn-in of 25%. Runs were stopped after checking that the average standard deviation of split frequencies reached a value below 0.01.

### Probe design and fluorescence *in situ* hybridization

Based on the obtained almost complete 16S rRNA gene sequences of bacterial symbionts, several probes were designed, targetting the whole “*Ca*. Megaira” genus, and for each of the most common clades A + D, B, C, and E, respectively (Table 2). Specificity was tested *in silico* both on RDP^35^ and on TestProbe tool 3.0 (SILVA rRNA database^60^) allowing 0 and 1 mismatches (Table 2). Probes were synthesized and labelled with Cy3 or fluorescein by Eurofins GMBH (Ebersberg, Germany).

The protocol used for all fluorescence *in situ* hybridization (FISH) experiments was the one described by Szokoli *et al*.^61^. Experiments always included negative controls, namely experiments without the use of any probe, and hybridizations were performed using several formamide concentrations in the hybridization buffer (0, 15 and 30% v/v) to test the different stringency levels for the newly designed FISH probes. The almost universal bacterial probe EUB338 (5′-GCTGCCTCCCGTAGGAGT-3′^62^) was used to verify the presence of other intracellular bacteria in the host cells.

### Transmission electron microscopy

*P*. *primaurelia* ThK-1, *P*. *caudatum* Sp 9-5, *P*. *bursaria* strains 1M-2^(T)^, VL3-1, VL12-10, *P*. *nephridiatum* Sr 2-6, and *P*. *putrinum* ETu 7-4 were prepared for electron microscopy as described by Nitla *et al*.^63^. Briefly, cells were fixed in 2.5% glutaraldehyde and 1.6% paraformaldehyde in phosphate buffer (0.1 M, pH 7.4), with a post-fixation in 1.5% OsO_4_. Afterwards cells were dehydrated at increasing percentages of ethanol solutions, and finally embedded in Epoxy embedding medium (Fluka, BioChemika). Ultrathin sections were stained with uranyl acetate followed by lead citrate. Samples were observed using JEOL JEM-1400 (JEOL, Ltd., Tokyo, Japan) electron microscope.

### Atomic force microscopy

*P*. *bursaria* VL12-10 cells were briefly washed in water, collected in a small drop, squashed on a cover slip and air-dried. The images were obtained with NTEGRA AURA microscope in a semi-contact mode.

### Screening of 16S rRNA gene datasets

Raw sequencing 16S rRNA gene reads derived from more than 111000 microbiome samples were screened using the platform IMNGS^33^, in order to use the results for investigating both diversity and environmental distribution of “*Ca*. Megaira” in the environments and in potential hosts. The almost complete 16S rRNA genes of Clade A - “*Ca*. Megaira polyxenophila” (Sp 11-8, AB688629), Clade B (HE648945, HE648946, FJ203077, DQ395479), Clade C - “*Ca*. Megaira venefica” (1M-2^(T)^, KT851791), Clade D (EU640051, KC189769), Clade E (KT851820, GQ870455), and the stand-alone sequence from wastewater (CU466797) were selected as representatives of “*Ca*. Megaira” genus and used as queries.

Sequences longer than 300 bp, and having at least 95% of identity with the queries were selected for analysis. Sequences retrieved from all queries were pooled together, and were separated in three datasets according to hypervariable regions V1-2, V4-6, V7-8, and then clustered in OTUs with a threshold at 99% using UCLUST^64^. Afterwards, OTUs were aligned with MUSCLE^65^, and phylogenetic analyses were performed using FastTree^66^. To attribute an environmental provenance to the OTUs, each sequence clustering in the same OTU was screened and assigned to its environment, according to the provenance of the original sample. Thus, a single environment was assigned to the respective OTU when more than 50% of sequences had the same origin, while two environments were considered when two ecosystems were equally dominant. The category “Unclassified” was used when more than two environments were dominant.

In order to further investigate environmental distribution of each “*Ca*. Megaira” clade (Clade A - “*Ca*. Megaira polyxenophila”, Clade B, Clade C - “*Ca*. Megaira venefica”, Clade D, and Clade E), two diverse indices were calculated: frequency of occurrence and relative abundance. Frequency of occurrence was estimated as the number of microbiome samples positive for “*Ca*. Megaira” in each category divided the total number of samples of the category (i.e. Freshwater; Seawater; Soil; Artificial/Anthropogenic including sequences associated to wastewater, food, activated carbon, bioreactor and activated sludge; Aquatic animals; Plants; Terrestrial animals). Relative abundance of “*Ca*. Megaira” for each specific environment was calculated as the average of relative abundances in “*Ca*. Megaira” positive samples (i.e. the number of “*Ca*. Megaira” reads divided total number of reads for each sample; for further details see Supplementary Text S3).

## Supplementary information

Supplementary Information

Supplementary Tables

Environmental distribution primary data
