# Supplementary material for: Diversity and environmental distribution of the cosmopolitan endosymbiont “Candidatus Megaira”
Source: Sci Rep. 2019 Feb 4;9:1179. doi: 10.1038/s41598-018-37629-w (PMC6362216; doi:10.1038/s41598-018-37629-w)
Supplement: Supplementary file 1 — Supplementary Information [file 41598_2018_37629_MOESM1_ESM.docx]

**Diversity and environmental distribution of the cosmopolitan endosymbiont “*Candidatus* Megaira”**

Olivia Lanzoni, Elena Sabaneyeva, Letizia Modeo, Michele Castelli, Natalia Lebedeva, Franco Verni, Martina Schrallhammer, Alexey Potekhin, Giulio Petroni.

**Supplementary Information**

**Supplementary Figure S1**

General view of an infected *Paramecium caudatum* strain Sp 4-1 cell infected both with “*Ca*. Megaira polyxenophila” in the macronucleus (MAC), and with *Holospora undulata* in the micronucleus (MIC).

**
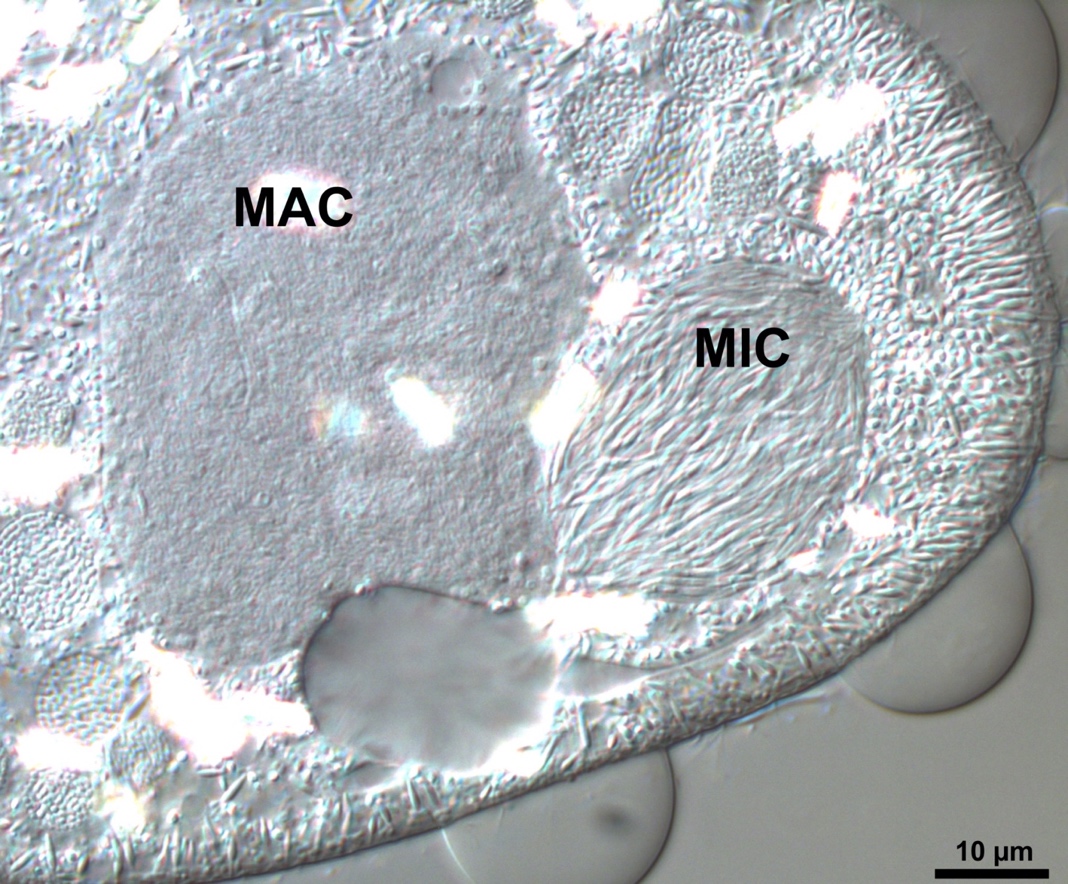
**

**Supplementary Figure S2**

Transmission electron microscopy of “*Ca.* Megaira venefica”. Transverse section of bacterial endosymbiont (black arrow) situated in the perialgal vacuole (white arrow) of the endosymbiotic algae of *P. bursaria* VL 3-1.


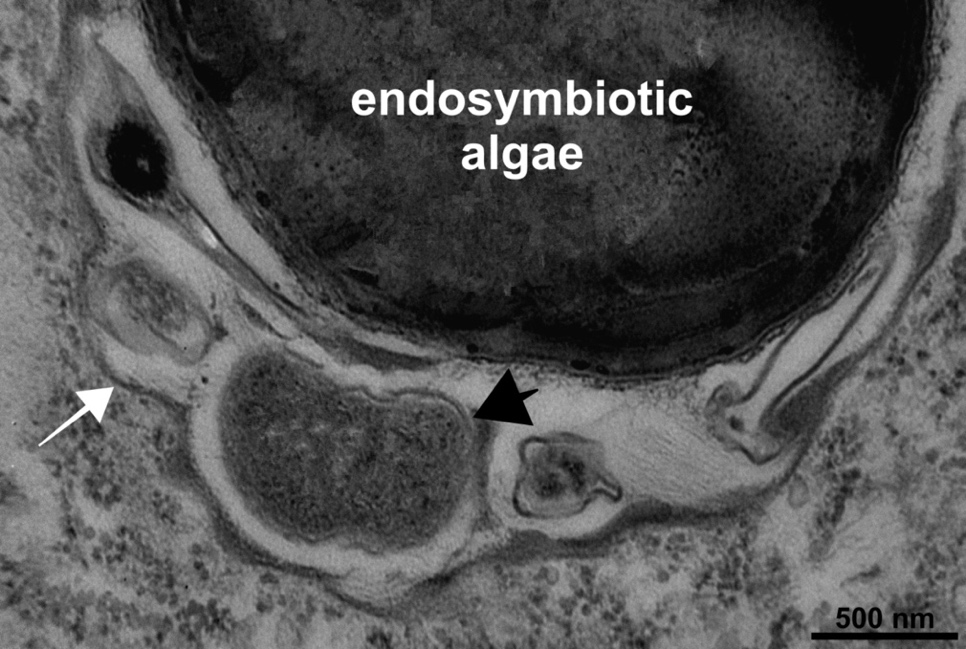


**Supplementary Text S1**

**Emended description of “*Candidatus* Megaira” Schrallhammer et al. (2013)**

“*Candidatus* Megaira” (Me.ga’i.ra. G. N. fem. “the Envious”, one of the Erinyes).

The type species is “*Ca*. Megaira polyxenophila” (Schrallhammer et al. 2013), and only another species is described “*Ca*. Megaira venefica” (present work). Basis of assignment: positive recognition with the FISH 16S rRNA targeting genus-specific probe Megenus_487 (5’-GCCGGGGCTTTTTCTGTTGGT-3’).

**Emended description of “*Candidatus* Megaira polyxenophila” Schrallhammer et al. (2013)**

“*Candidatus* Megaira polyxenophila” (po.ly.xe.no’.phi.la, G. adj. polýs, many, G. N. xénos, host, G. v. phıleîn, to love, which loves many hosts).

Bacteria have a typical Gram-negative structure and are rod-shaped. Size is variable between 4.8 µm and 1.5 µm in length and 0.5 µm in width (average size 1.5 µm x 0.5 µm). Bacteria were never observed surrounded by additional membranes and often are surrounded by a halo. The microorganism can be found in different hosts, namely ciliates, where it is localized both in the macronucleus (*Paramecium caudatum*, *Spirostomum* sp.) or in the cytoplasm (*Paramecium aurelia*, *Colpidium striatum*, *Diophrys* sp., *Euplotes* sp.), but also in Archaeplastida cytoplasm (*Carteria*, *Pleodorina*). The bacterium is a member of the *Alphaproteobacteria*, *Rickettsiales*, *Rickettsiaceae*, “*Candidatus* Megaira”. Its classification is based on 16S rRNA gene sequences and fluorescence *in situ* hybridization with the probe MegPoly_66 (5’-GCAAGCCCCAATTTTGTTCGT-3’). Type strain: endosymbiont of *Diophrys oligothrix* BOD9 (AJ630204) where the symbiont was firstly described. Uncultured thus far.

**Description of “*Candidatus* Megaira venefica” sp. nov.**

“*Candidatus* Megaira venefica” (ve.ne’fi.ca, L. adj. fem. charmer that evokes fascination). This name was chosen as for a certain period of our research this symbiont was retrieved in every new analyzed *Paramecium* strain.

Bacteria are rod-shaped and of variable size, generally measuring 1.9 µm x 0.5 µm, and show a typical Gram-negative cell wall. An electron translucent zone (halo) often surrounds cells. Intracellular bacteria are located in the cytoplasm of several species of *Paramecium*, namely *P*. *bursaria*, *P*. *nephridiatum*, *P*. *putrinum*. It can be present inside the perialgal vacuole, and flagella can be sometimes observed. The microorganism is a member of the *Alphaproteobacteria*, *Rickettsiales*, *Rickettsiaceae*, “*Candidatus* Megaira”. Its classification is based on 16S rRNA gene sequences and fluorescence *in situ* hybridization with the probe MegVene_95 (5’-CCGTTTGCCACTAACGAC-3’). Type strain: endosymbiont of *P. bursaria* 1M-2 (MG563925) where the symbiont was firstly discovered and characterized. Uncultured thus far.

**Supplementary Text S2**

**Material & Methods of molecular characterization**

| **Target** | **Primer name** | **Sequence (5’-3’)** | **Application** | **Reference** |
| --- | --- | --- | --- | --- |
| **16S rRNA gene** |  |  |  |  |
|  | 16S alfa F19a | CCTGGCTCAGAACGAACG | PCR | Vannini et al. 2004 |
|  | R1492 | GGNWACCTTGTTACGACTT | PCR | Lane et. 1991 |
|  | 16S alfa R1517 | TGATCCAGCCGCAGGTTC | PCR | Vannini et al. 2004 |
|  | Bac 16S F7a | AGAGTTTGATCCTGGCTCAG | PCR | Vannini et al. 2004 |
|  | R1522 | GGAGGTGATCCADCCDCA | PCR | Lane et. 1991 |
|  | Bac R515 | ACCGCGGCTGCTGGCAC | Sequencing | Vannini et al. 2004 |
|  | Bac F343 | TACGGGAGGCAGCAG | Sequencing | Vannini et al. 2004 |
|  | Bac F785 | GGATTAGATACCCTGGTA | Sequencing | Vannini et al. 2004 |
|  | F487_Meg | ACCAACAGAAAAAGCCC | Sequencing | This study |
|  | R507_Meg | GCCGGGGCTTTTTCTGTT | Sequencing | This study |
| **18S rRNA gene** |  |  |  |  |
|  | 18S F9 Euk | CTGGTTGATCCTGCCAG | PCR | Medlin et al. 1988 |
|  | 18S R1513 Hypo | TGATCCTTCYGCAGGTTC | PCR | Petroni et al. 2002 |
|  | 18S R536 | CTGGAATTACCGCGGCTG | Sequencing | Rosati el. 2004 |
|  | 18S F300 | AGGGTTCGATTCCGGAGA | Sequencing | Rosati el. 2004 |
|  | 18S F783 | GACGATCAGATACCGTC | Sequencing | Rosati el. 2004 |
|  | Chlo F59 | CATGTCTAAGTATAAACTGCT | PCR | Lanzoni et al. 2016 |
|  | Chlo R1052 | CCTGACAAGGCAACCC | PCR | Lanzoni et al. 2016 |
|  | Chlo F194a | TATTAGATAAAAGGCCGACC | Sequencing | Lanzoni et al. 2016 |
|  | Chlo R426 | CTCATTCCAATTACCAGAC | Sequencing | Lanzoni et al. 2016 |
|  | Chlo F770 | TGGGGGCTCGAAGAC | Sequencing | Lanzoni et al. 2016 |
| **COI** |  |  |  |  |
|  | F388dT | TGTAAAACGACGGCCAGTG  GWKCBAAAGATGTWGC | PCR & Sequencing | Strüder-Kypke et al. 2000 |
|  | R1184dT | CAGGAAACAGCTATGACTADACY  TCAGGGTGACCRAAAAATCA | PCR & Sequencing | Strüder-Kypke et al. 2000 |
| **ITS1-5.8S-ITS2** |  |  |  |  |
|  | 18S F919 | ATTGACGGAAGGGCACCA | PCR | Rosati et al. 2004 |
|  | RGD2 | GGTCCGTGTTTCAAGACGGG | PCR & Sequencing | Boscaro et al. 2012 |
|  | FG1400 | TTGYACACACCGCCCGTC | Sequencing | Boscaro et al. 2012 |

**References:**

Vannini, C., Rosati, G., Verni, F., & Petroni, G. Identification of the bacterial endosymbionts of the marine ciliate *Euplotes magnicirratus* (Ciliophora, Hypotrichia) and proposal of “*Candidatus* Devosia euplotis”. *Int J Syst Evol Micr*, **54,** 1151-1156 (2004).

Lane, D. J. 16S/23S rRNA sequencing. *Nucleic acids techniques in bacterial systematics*, 115-175 (1991).

Medlin, L., Elwood, H.J., Stickel, S., Sogin, M.L. The characterization of enzymatically amplified 16S-like rRNA-coding regions. *Gene*, **71**: 491–499 (1988).

Petroni, G., Dini, F., Verni, F., Rosati, G. A molecular approach to the tangled intrageneric relationships underlying phylogeny in *Euplotes* (Ciliophora, Spirotrichea). *Molecular Phylogenetics and Evolution*, **22**: 118–130 (2002).

Rosati, G., Modeo, L., Melai, M., Petroni, G., & Verni, F. A Multidisciplinary Approach to Describe Protists: a Morphological, Ultrastructural, and Molecular Study on *Peritromus kahli* (Ciliophora, Heterotrichea). *J Eukaryot Microbiol*, **51,** 49-59. (2004).

Lanzoni, O. *et al*. Rare Freshwater Ciliate *Paramecium chlorelligerum* Kahl, 1935 and Its Macronuclear Symbiotic Bacterium “*Candidatus* Holospora parva”. *PloS One*, **11,** e0167928 (2016).

Strüder-Kypke, M.C., Wright, A.D.G., Fokin, S.I, Lynn, D.H. Phylogenetic relationships of the genus *Paramecium* inferred from small subunit rRNA gene sequences. *Molecular Phylogenetics and Evolution*, **14**: 122–130 (2000).

Boscaro, V., Fokin, S. I., Verni, F., & Petroni, G. Survey of *Paramecium duboscqui* using three markers and assessment of the molecular variability in the genus *Paramecium*. *Molecular phylogenetics and evolution*, **65**: 1004-1013 (2012).

**Supplementary Text S3**

**Material & Methods of environmental distribution**

A total number of 111525 samples derived from IMNGS research was analyzed and assigned to a specific environment or host. Samples from environments were classified in: Freshwater (all samples assigned as freshwater, riverine, lake, aquatic), Seawater (all marine, seawater, hypersaline lake, hydrothermal vent samples), Artificial (wastewater, food, activated carbon, bioreactor, activated sludge samples), and Soil (soil, terrestrial, sediment, sand samples).

In case of hosts three categories were created: Aquatic organisms (which included Porifera, fish, Cnidaria, Crustacea, echinoderm, mollusks), Terrestrial organisms (which comprised insects, Nematoda, other Vertebrata), and Plants. All samples which did not have a clear and precise indication of their provenance were discarded.
